# Activating Fc Gamma Receptors and Viral Receptors Are Required for Antibody-Dependent Enhancement of Porcine Reproductive and Respiratory Syndrome Virus Infection

**DOI:** 10.3390/vetsci9090470

**Published:** 2022-08-31

**Authors:** Liujun Zhang, Huandi Wang, Wen Li, Xing Feng, Fangfang Han, Yina Zhang, Jing Chen, Deyi Liu, Pingan Xia

**Affiliations:** 1 College of Animal Science, Anhui Science and Technology University, Chuzhou 233100, China; 2 College of Veterinary Medicine, Henan Agricultural University, Zhengzhou 450046, China

**Keywords:** PRRSV, ADE, FcγRs, Sn, CD163

## Abstract

**Simple Summary:**

Porcine reproductive and respiratory syndrome virus (PRRSV)-specific sub- or non-neutralizing antibodies promote the adhesion and internalization of the virion into host cells. This phenomenon is known as antibody-dependent enhancement (ADE) of PRRSV infection. It has long been accepted that Fc gamma receptors (FcγRs) are responsible for mediating ADE of virus infection. However, few researchers pay attention to the role of the virus receptors in the ADE of virus infection. In this study, we showed that activating FcγRs (FcγRI and FcγRIII) were responsible for mediating PRRSV-ADE infection. Simultaneously, we showed that the viral receptors (sialoadhesin and CD163) were involved in FcγR-mediated PRRSV-ADE infection. The extracellular domains 1-6 of sialoadhesin and the scavenger receptor cysteine-rich 5 domain of CD163 might play central roles in PRRSV-ADE infection. In conclusion, our studies indicated that activating FcγRs and virus receptors were required for PRRSV-ADE infection. Our findings should allow a more precise understanding of the structural basis for the mechanism of PRRSV-ADE infection, which would provide references for screening targets of novel PRRS vaccines or antiviral drugs against the PRRSV.

**Abstract:**

Antibody-dependent enhancement (ADE) is an event in preexisting sub-, or non-neutralizing antibodies increasing the viral replication in its target cells. ADE is one crucial factor that intensifies porcine reproductive and respiratory syndrome virus (PRRSV) infection and results in PRRSV-persistent infection. Nevertheless, the exact mechanisms of PRRSV-ADE infection are poorly understood. In the current research, the results of the ADE assay showed that porcine immunoglobulin G (IgG) specific for the PRRSV significantly enhanced PRRSV proliferation in porcine alveolar macrophages (PAMs), suggesting that the ADE activity of PRRSV infection existed in pig anti-PRRSV IgG. The results of the RNA interference assay showed that knockdown of the Fc gamma receptor I (FcγRI) or FcγRIII gene significantly suppressed the ADE activity of PRRSV infection in PAMs, suggesting that FcγRI and FcγRIII were responsible for mediating PRRSV-ADE infection. In addition, the results of the antibody blocking assay showed that specific blocking of the Sn1, 2, 3, 4, 5, or 6 extracellular domain of the sialoadhesin (Sn) protein or selective blockade of the scavenger receptor cysteine-rich (SRCR) 5 domain of the CD163 molecule significantly repressed the ADE activity of PRRSV infection in PAMs, suggesting that Sn and CD163 were involved in FcγR-mediated PRRSV-ADE infection. The Sn1–6 domains of porcine Sn protein and the SRCR 5 domain of porcine CD163 molecule might play central roles in the ADE of PRRSV infection. In summary, our studies indicated that activating FcγRs (FcγRI and FcγRIII) and viral receptors (Sn and CD163) were required for ADE of PRRSV infection. Our findings provided a new insight into PRRSV infection that could be enhanced by FcγRs and PRRSV receptors-mediated PRRSV-antibody immune complexes (ICs), which would deepen our understanding of the mechanisms of PRRSV-persistent infection via the ADE pathway.

## 1. Introduction

Since first described three decades ago in the United States, porcine reproductive and respiratory syndrome (PRRS) has been arguably one of the most economically significant highly contagious diseases heavily impairing pigs around the globe [1]. The etiologic agent is the RNA enveloped PRRS virus (PRRSV) in the *Arteriviridae* family. PRRSV isolates are genetically grouped into two distinct species, the European PRRSV and North American PRRSV [2,3]. Both subtypes of PRRSV are now extensively distributed and cause abortions, late-term stillbirths, mummies, high mortality rates, poor growth, and respiratory problems in pigs, incurring substantial economic losses in worldwide swine production [4]. PRRSV has restricted host and cell tropism for the monocyte/macrophage lineage. The porcine alveolar macrophages (PAMs) act as the principal cell targets for PRRSV replication. Moreover, the Marc-145 cells support PRRSV growth in vitro [5]. Even though PRRSV infection induces rapid humoral immune responses to produce a mass of specific antibodies, these early antibody responses correspond to sub- or non-neutralizing antibodies. The inadequate humoral immune responses cannot eliminate the virus from the hosts. On the contrary, PRRSV depends on these sub- or non-neutralizing antibodies for its invasion into host cells such as macrophages, monocytes, and dendritic cells (DCs). This phenomenon is called antibody-dependent enhancement (ADE) [6,7]. ADE not only promotes PRRSV entry into the host cells but also down-modulates the host antiviral immunity and causes the poor effectiveness of vaccination, thereby leading to PRRSV-persistent infection in swine herds [8,9,10]. Furthermore, ADE is also considered the main obstacle to developing an efficacious PRRS vaccine [11]. Dissection of the ADE event will contribute to understanding the pathogenesis of PRRSV and the rational design of novel PRRS vaccines.

Receptors for the constant Fc domain (FcγRs) of the immunoglobulin G (IgG) are broadly expressed on the surface of most effector leukocytes throughout the hematopoietic systems, such as monocytes/macrophages, natural killer cells, DCs, B cells, and other immune cells [12]. Based on their ability to recognize and bind the two primary conformational states of the Fc portion of the IgG molecule, FcγRs have two basic types, type I and type II. There are four kinds of FcγRs in type I receptors of humans and mammal animals: FcγRI, FcγRII, FcγRIII, and FcγRIV [13]. FcγRI displays a relatively high affinity for the IgG Fc domain and is able to interact effectively with monomeric IgG. FcγRII and FcγRIII are low-affinity protein molecules and are only occupied by the IgG-containing immune complexes (ICs). FcγRIV is an intermediate affinity receptor that can bind to IgG2a and IgG2b [14,15]. All Fc receptors can mediate ADE of several virus infection, including zika virus, dengue virus, Ebola virus, influenza virus, severe acute respiratory syndrome coronavirus-2 (SARS-CoV-2), Ross River virus, West Nile virus, enterovirus, and human immunodeficiency virus type 1 (HIV-1) [16,17,18]. So far, porcine type I FcγRs (FcγRI, FcγRII, and FcγRIII) have been successively cloned and characterized. Of three classes of FcγRs, porcine FcγRI and FcγRIII are the activating receptors belonging to a typical transmembrane (TM) glycoprotein with an ectocytic peptide followed by a hydrophobic domain and an endocellular region lack of known signaling transduction motifs [19,20]. However, their roles in the ADE of virus infection have not yet been well investigated.

Sialoadhesin (Sn), the first identified Siglec member in the Ig superfamily, has the typical features of a type I TM glycoprotein composed of an extracellular portion of seventeen Ig-like domains, a TM fragment, and an intracellular segment [21,22]. Sn can participate in endocytosis, antigen presentation, cell–cell interactions, activation of adaptive immunity, inhibition of innate immune responses, and immune escape of HIV-1 [23,24]. Sn also serves as a cellular receptor to capture and internalize important pathogenic microbes into their host cells, such as PRRSV, HIV-1, Nipah virus, Hendra hemorrhagic fever virus, and so on [25,26,27]. CD163 molecule is a member of the scavenger receptor cysteine-rich (SRCR) protein superfamily and is exclusively expressed on the cytomembrane of macrophages. CD163 molecule also belongs to type I TM glycoprotein, consisting of a sizeable ectocytic region, a TM fragment, and a cytosolic tail [28]. Its extracellular area is composed of nine tandem repeat SRCR domains numbered 1–9. The SRCR domain is an ancient extracorpuscular domain containing about one hundred amino acid residues [29]. CD163 molecule is capable of regulating the immune, anti-inflammatory, or inflammatory responses, and internal homeostatic balance, by recognizing and binding the host molecules and pathogens, including polyribonucleotides, proteins, polysaccharides, lipids, and so on [30]. For instance, binding the hemoglobin/haptoglobin complexes or bacteria to the CD163 mediates the interleukin-10 synthesis, which induces the CD163 and heme enzyme production [31]. In addition, CD163 is a critical molecule in PRRSV entry into PAMs [32].

Although porcine FcγRs, Sn, and CD163 molecules have been identified for many years, their roles in ADE of PRRSV infection are not well understood. We reported the effects of porcine FcγRI, FcγRIII, Sn, and CD163 on ADE of PRRSV infection here. The results would facilitate an understanding of the interaction between PRRSV-antibody immune ICs, FcγRs, and viral receptors.

## 2. Materials and Methods

### 2.1. Cells and Virus

PAMs used in this study were separated by the bronchoalveolar lavage of the lungs of PRRSV-negative piglets of 4 to 6 weeks old as previously described [33] and then kept in the RPMI-1640 medium (HyClone, Logan, UT, USA) containing 10% heat-inactivated fetal bovine serum (FBS; HyClone), 100 U/mL penicillin (HyClone), and 100 μg/mL streptomycin (HyClone). Marc-145 were routinely cultivated in the DMEM medium (HyClone) with 10% heat-inactivated FBS at 37 °C in 5% CO_2_. North American PRRSV used for all experiments was isolated from PAMs and titrated for the 50% tissue culture infectious dose (TCID_50_).

### 2.2. Antibodies

The inactivated purified PRRSV particles were used to immunize the swine for the generation of the PRRSV-specific polyclonal antibody (pAb) (enzyme-linked immunosorbent assay (ELISA) titer: 5200). FcγRI-specific pAb (ELISA titer: 12,800) was from the rabbits inoculated with the recombinant proteins of porcine FcγRI extracellular domain. FcγRIII-specific pAb (ELISA titer: 12,800) was from the rabbits immunized with the recombinant proteins of porcine FcγRIII extracellular domain. The extracellular N-terminal domains of porcine Sn protein (shown in Table 1) and the ectocytic SRCR domains of porcine CD163 molecule (described in Table 2) were analyzed using the SMART online analysis service based on each protein sequence deposited in NCBI (GenBank ID: NP999511; EU016226.1). The pAb specific for the extracellular domain of Sn1, 2, 3, 4, 5, 6, 7, 8, 9, or 1–9 (ELISA antibody titers: 6400–12,800) was gained from the rabbits immunized with recombinant proteins of porcine Sn1 (extracellular domain 1), 2 (extracellular domain 2), 3 (extracellular domains 3 and 4), 4 (extracellular domains 5 and 6), 5 (extracellular domains 7 and 8), 6 (extracellular domains 9 and 10), 7 (extracellular domains 11 and 12), 8 (extracellular domains 13 and 14), 9 (extracellular domains 15, 16, and 17), or 1–9 (extracellular domains 1 to 17), respectively. The pAb specific for the CD163 of SRCR1, 2, 3, 4, 5, 6, 7, 8, 9, or 1–9 (ELISA antibody titers: 6400–12,800) was obtained from the rabbits immunized with purified proteins of porcine CD163 SRCR1, 2, 3, 4, 5, 6, 7, 8, 9, or 1–9 (SRCR domains 1 to 9), respectively. PRRSV-specific IgG, FcγRI-specific IgG, FcγRIII-specific IgG, Sn extracellular domain-specific IgG, or CD163 SRCR domain-specific IgG was purified by diethyl-aminoethanol chromatography. PRRSV-negative IgG (PNI) was purified from PRRSV-negative piglet sera. Rabbit-negative IgG (RNI) was purified from healthy rabbit serums. Anti-rabbit IgG antibody linked with horseradish peroxidase (HRP) or fluorescein isothiocyanate (FITC) and anti-glyceraldehyde-3-phosphate dehydrogenase (GAPDH) antibody labeled by HRP were the products of the Cell Signaling Technology in the USA.

### 2.3. Formation of PRRSV-Antibody ICs

PRRSV-specific IgG or PRRSV-negative IgG (PNI) was diluted to 850 μg/mL. Then, 2000 TCID_50_/mL of PRRSV suspensions were mixed with 850 μg/mL of PRRSV-specific IgG or PNI in equal volumes for one hour at 37 °C for the formation of the infectious PRRSV-antibody ICs (marked as PRRSV + ICs) or the negative control group (flagged as PRRSV + PNI).

### 2.4. Detection of PRRSV-ADE Infection

PAMs cell monolayer (5 × 10^5^ cells) cultured in 24-well plates (Corning, NY, USA) was infected with 200 μL of PRRSV + PNI, PRRSV + ICs, or PRRSV containing 200 TCID_50_ at 37 °C. The cell supernatants were harvested in indicated time point to quantify viral RNA as previously reported real-time RT-PCR [34] and measure virus titers using the Reed–Muench method.

### 2.5. RNA Interfering Assay

The negative control small interfering RNA (siRNA) and the siRNA used for silencing porcine FcγRI or FcγRIII gene (seen in Table 3) were supplied by the Shanghai GenePharma Corporation in China. 20 pmol of negative control siRNA, FcγRI siRNA, or FcγRIII siRNA was transfected into PAMs cell monolayer (5 × 10^5^ cells) prepared aforehand in 24-well plates by utilizing lipofectamine 2000 reagent (Invitrogen, Beijing, China) for knockdown of the target genes. Between 24–72 h later, the transfected cells were gathered for quantitative RT-PCR, immunoblot, or flow cytometry. Moreover, 48 h post-transfection, 200 μL PRRSV + ICs were used to infect the transfected cells for 12 and 24 h at 37 °C. Then the cell supernatants were harvested for detection of viral RNA and titers.

### 2.6. Antibody Blocking Assay

PAMs cell monolayer (5 × 10^5^ cells) seeded in 24-well plates were pre-blocked with 200 μL of each rabbit anti-Sn and anti-SRCR domain IgG or rabbit-negative IgG (RNI) at 2.0 mg/mL concentration before they were infected with 200 μL PRRSV + ICs. The cell supernatants were harvested in indicated time point for the determination of viral RNA and titers.

### 2.7. RNA Extraction and Quantification RT-PCR

Total PAMs RNAs were isolated with the TRIzol reagent (TaKaRa, Dalian, China). The RNAs were reverse-transcribed into cDNA using the commercialized reagent kit (TaKaRa) and then subjected to the analysis of relative quantitative RT-PCR with the Bio-Rad’s CFX 96 Touch System by previously described [27] using specific primer pairs presented in Table 4. The target gene quantification was analyzed with the 2^−^^∆∆CT^ method.

### 2.8. Immunoblot Assay

The primary rabbit anti-FcγRI and anti-FcγRIII IgG (20 μg/mL) and the secondary anti-rabbit IgG antibody (HRP-linked) (1:3000 dilution) were used for the immunoblot experiment. PAMs cells collected from each sample were lysed, and the cellular proteins were separated by 10% sodium dodecyl sulfate-polyacrylamide gel electrophoresis and transferred onto a polyvinylidene difluoride (PVDF) membrane (Sigma, Saint Louis, MO, USA). After blocking, the PVDF membrane was probed with the indicated primary and secondary antibodies. The reference protein was detected with HRP-labeled anti-GAPDH antibody (1:1000 dilution). The immunolabeled target proteins were visualized with the GE Healthcare’s ECL chemiluminescence reagent.

### 2.9. Flow Cytometry

The primary rabbit anti-FcγRI or anti-FcγRIII IgG and the secondary anti-rabbit IgG antibody (FITC-conjugated) were used for the flow cytometry experiment. At 37 °C, PAMs collected were pretreated with rabbit-negative IgG (RNI), rabbit anti-FcγRI IgG, or rabbit anti-FcγRIII IgG at 100 μg/mL concentration for one hour and then incubated with anti-rabbit IgG antibody (1:100 dilution) for one hour. In each step, the cells were washed using PBS buffer containing 3% FBS. A CytoFLEX flow cytometer from the USA Beckman Coulter was used to perform flow cytometry analysis.

### 2.10. Statistical Analysis

The two-way analysis of variance in GraphPad Prism software (Version 5.0) was used for analyzing data, and the *p* values < 0.05 were considered significant.

## 3. Results

### 3.1. ADE Activity of PRRSV Infection Exists in PRRSV-Specific Porcine IgG in PAMs

**Figure 1:**
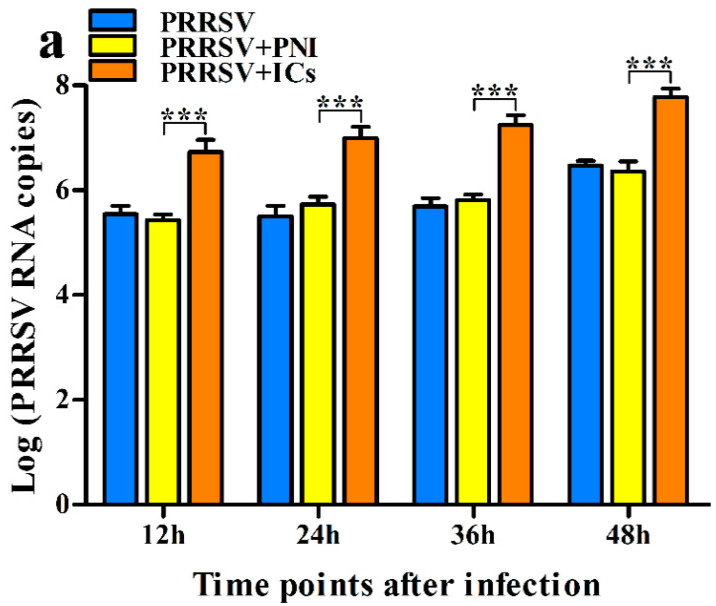

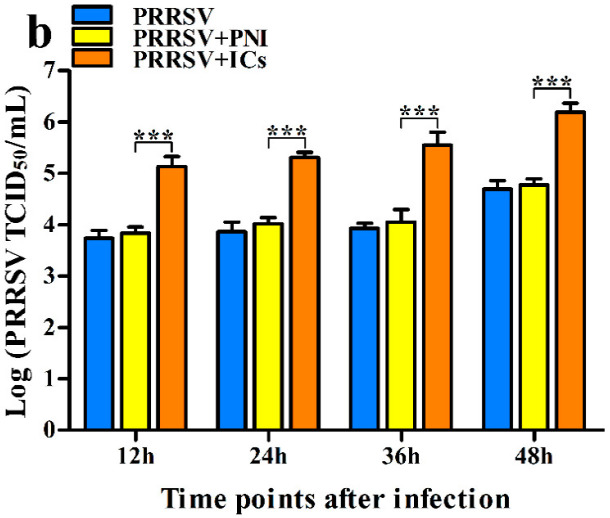
Effect of pig anti-PRRSV specific IgG on the replication of PRRSV in PAMs. PAMs cells were infected with PRRSV + ICs, PRRSV + PNI, or PRRSV. The RNA levels (**a**) and the virus titers (**b**) of PRRSV in cell supernatants harvested were evaluated by real-time RT-PCR and TCID_50_ assay. *** *p* < 0.001.

To determine whether pig anti-PRRSV specific IgG could affect PRRSV replication, PAMs cells were infected with PRRSV + ICs or PRRSV + PNI. Then, we collected the cell supernatants at different time points for the detection of the production of PRRSV. As shown in Figure 1, at any time point after infection, the levels of RNA and titers of PRRSV in supernatants of PAMs cells following PRRSV + ICs infection were significantly more than that in supernatants of PAMs cells following PRRSV + PNI infection, suggesting that PRRSV-specific porcine IgG enhanced the replication of PRRSV in PAMs cells. In other words, ADE activity of PRRSV infection existed in PRRSV-specific porcine IgG.

### 3.2. FcγRI and FcγRIII Are Responsible for Mediating ADE of PRRSV Infection in PAMs

**Figure 2:**
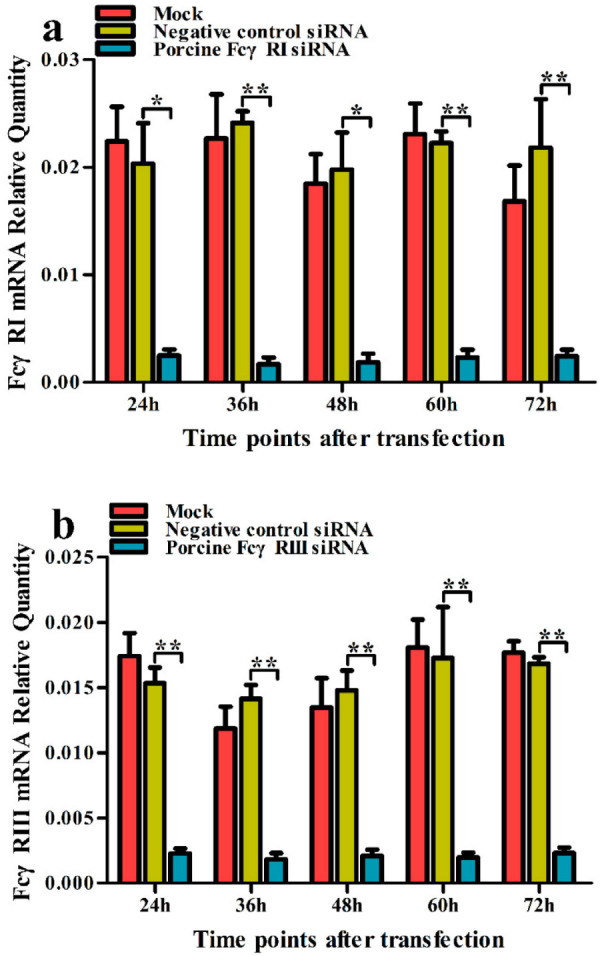
Analysis of the transcription expression of FcγRI or FcγRIII in PAMs. PAMs cells were transfected with porcine FcγRI or FcγRIII siRNA. The mRNA of FcγRI (**a**) or FcγRIII (**b**) in transfected cells was measured by relative quantitative RT-PCR. ** *p* < 0.01, * *p* < 0.05.

**Figure 3:**
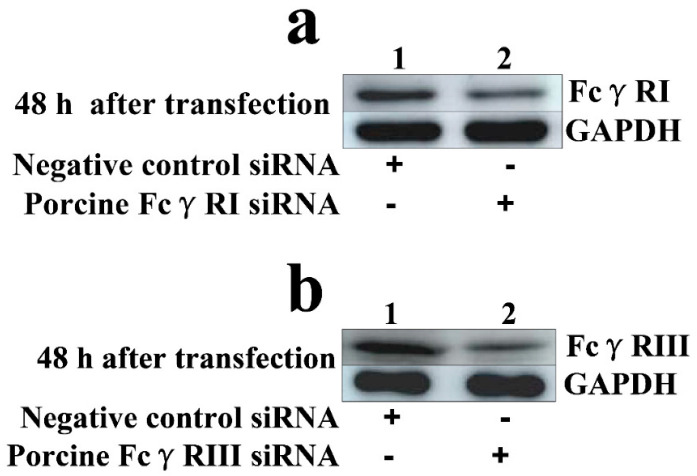
Analysis of the total cellular protein expression of FcγRI or FcγRIII in PAMs. PAMs cells were transfected with porcine FcγRI or FcγRIII siRNA for forty-eight hours and then used for the analysis of immunoblot. The total cellular protein level of FcγRI (**a**) or FcγRIII (**b**) in transfected cells was quantified by Western blot. The original version of the Western blot can be seen in Supplementary Materials. The GAPDH was used as a reference protein.

**Figure 4:**
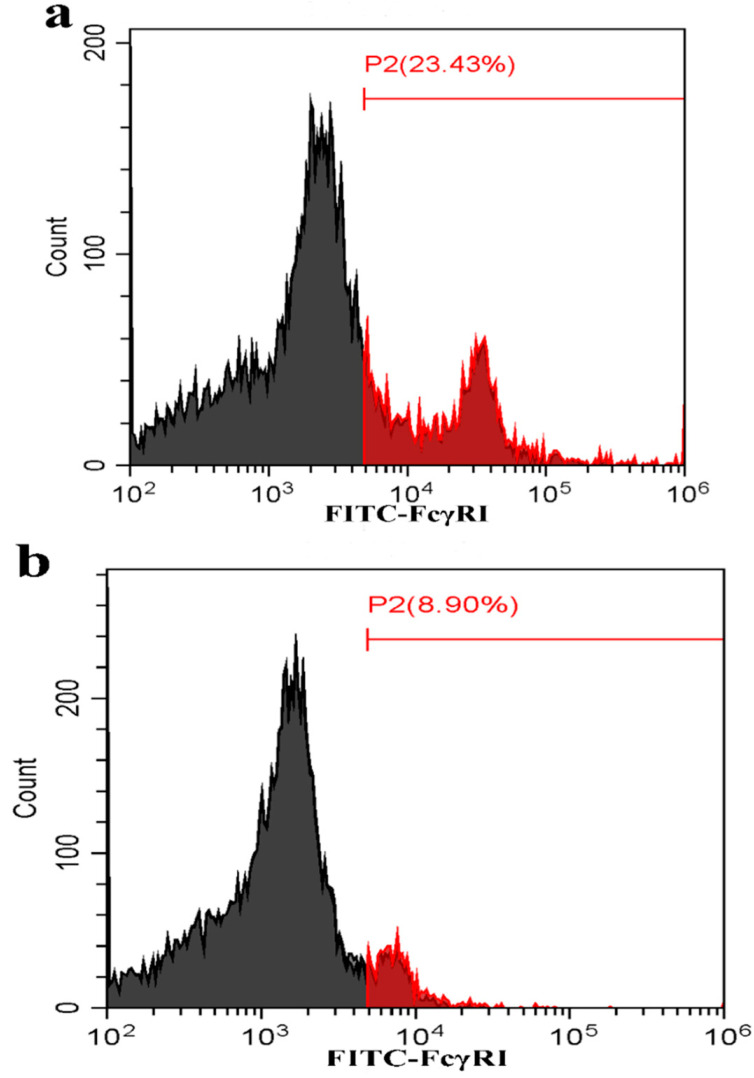

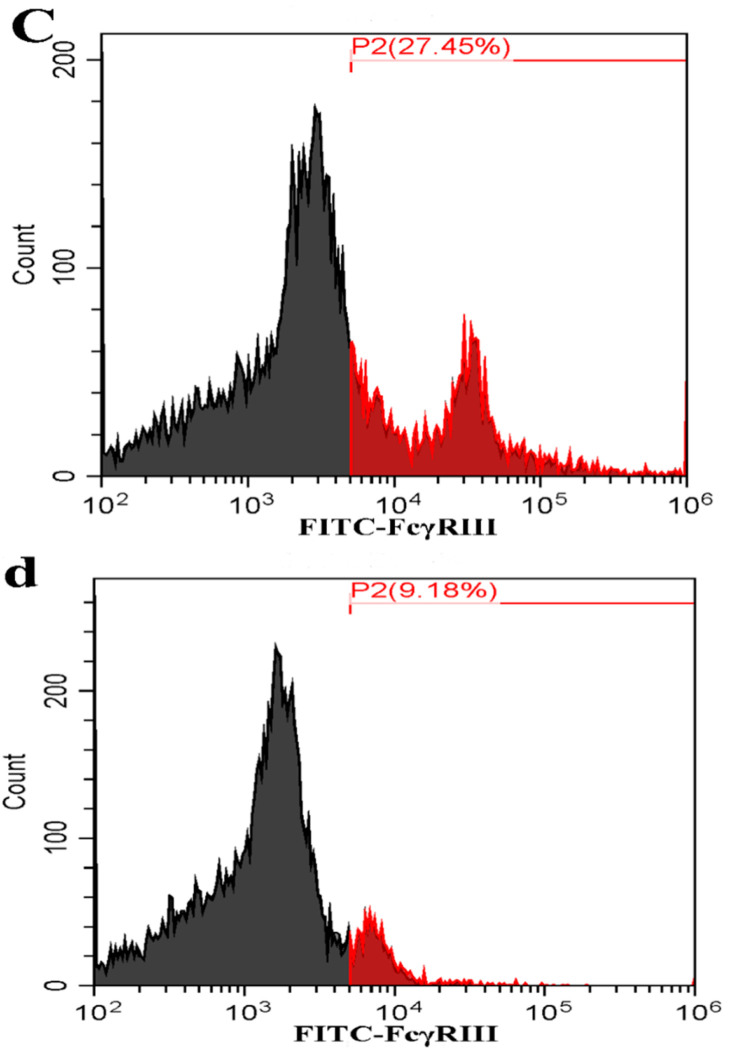
The cytomembrane protein level of FcγRI or FcγRIII on PAMs. PAMs cells were transfected with porcine FcγRI or FcγRIII siRNA for forty-eight hours and then used for the analysis of flow cytometry. (**a**) The cytomembrane protein level of FcγRI on the PAMs cells following negative control siRNA transfection; (**b**) The cytomembrane protein level of FcγRI on the PAMs cells following porcine FcγRI siRNA transfection; (**c**) The cytomembrane protein level of FcγRIII on the PAMs cells following negative control siRNA transfection; (**d**) The cytomembrane protein level of FcγRIII on the PAMs cells following porcine FcγRIII siRNA transfection.

**Figure 5:**
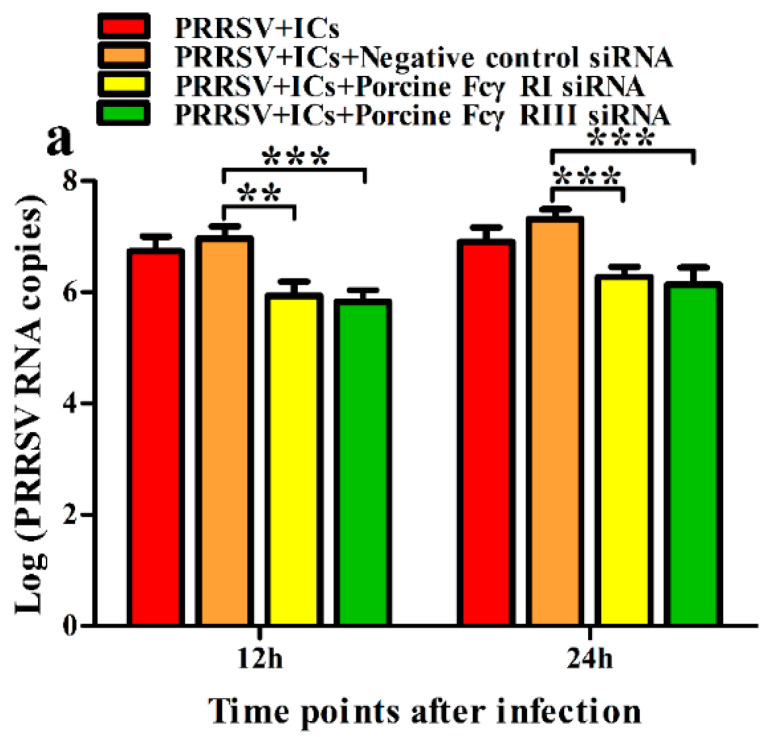

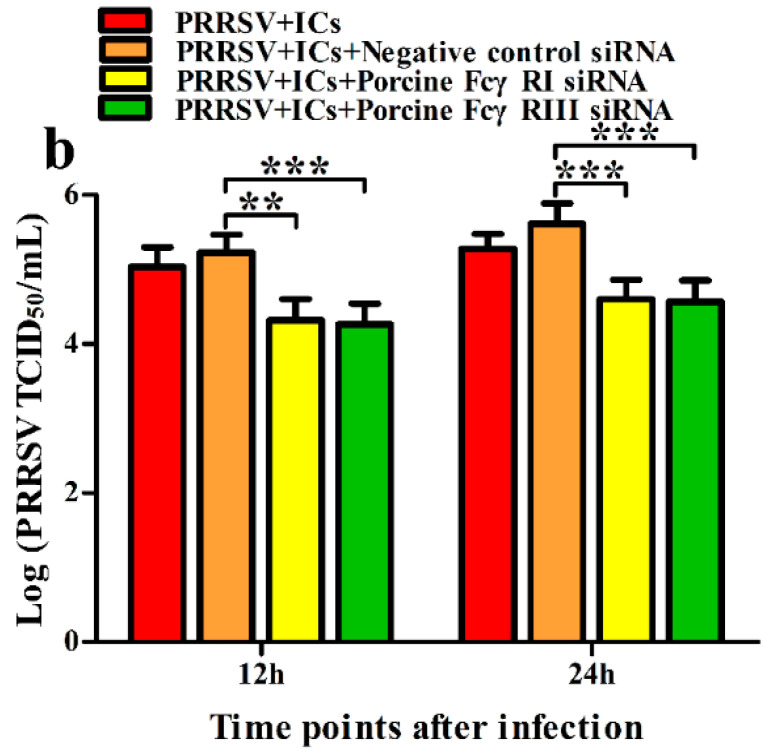
Effect of FcγRI or FcγRIII knockdown on ADE activity of PRRSV infection in PAMs. PAMs cells were transfected with porcine FcγRI or FcγRIII siRNA for forty-eight hours and then infected by PRRSV + ICs for the indicated time. The RNA levels (**a**) and the virus titers (**b**) of PRRSV in cell supernatants harvested were evaluated by real-time RT-PCR and TCID_50_ assay. *** *p* < 0.001, ** *p* < 0.01.

To test whether activating FcγRs were responsible for mediating PRRSV-ADE infection, PAMs cells were transfected with porcine FcγRI or FcγRIII siRNA for indicated time points, and forty-eight hours later the cells were infected by PRRSV + ICs. Twelve and twenty-four hours later, the production of PRRSV in infected cell supernatants harvested was quantified. The results seen in Figure 2 and Figure 3 showed that transfection with porcine FcγRI siRNA or porcine FcγRIII siRNA into PAMs cells caused significant downregulation of porcine FcγRI mRNA or porcine FcγRIII mRNA at 24–72 h after transfection, and porcine FcγRI protein or porcine FcγRIII protein at forty-eight hours after transfection, compared to PAMs cells following negative control siRNA transfection. Additionally, transfection with porcine FcγRI siRNA or porcine FcγRIII siRNA into PAMs cells also led to a visible reduction in porcine FcγRI protein or porcine FcγRIII protein on the surface of transfected cells at forty-eight hours after transfection, compared to negative control siRNA-transfected cells (Figure 4). These results suggested that siRNA targeting porcine FcγRI or FcγRIII resulted in knockdown of FcγRI or FcγRIII gene in PAMs cells. Simultaneously, the results depicted in Figure 5 showed that FcγRI or FcγRIII knockdown in PAMs cells significantly decreased the RNA and the titers of PRRSV in collected supernatants of the cells following PRRSV + ICs infection for 12 and 24 h, which suggested that porcine FcγRI and FcγRIII molecules were responsible for mediating ADE of PRRSV infection.

### 3.3. Sn and CD163 Are Required for ADE of PRRSV Infection in PAMs

**Figure 6:**
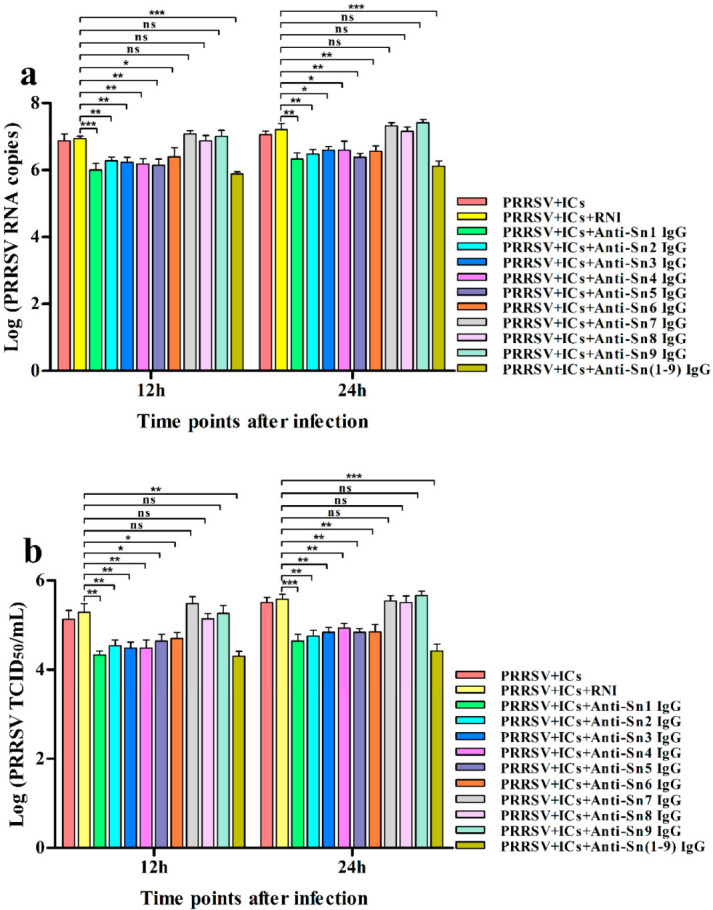
Effect of the antibody blocking of Sn extracellular domains on PRRSV-ADE activity in PAMs. PAMs cells were pre-blocked by each rabbit anti-Sn domain IgG or RNI and then infected with PRRSV + ICs. The RNA levels (**a**) and the virus titers (**b**) of PRRSV in cell supernatants harvested were evaluated by real-time RT-PCR and TCID_50_ assay. *** *p* < 0.001, ** *p* < 0.01, * *p* < 0.05, ns: no significance.

**Figure 7:**
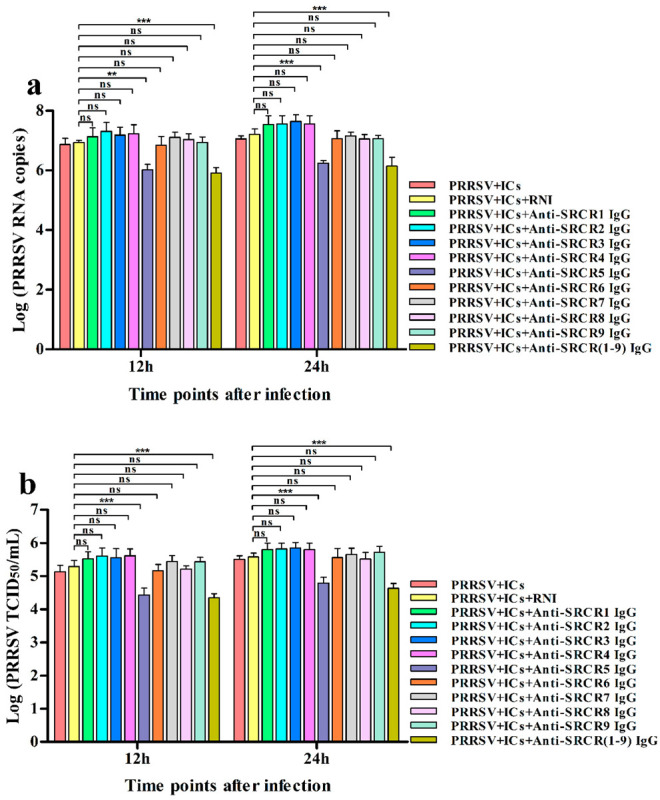
Effect of the antibody blocking of CD163 SRCR domains on PRRSV-ADE activity in PAMs. PAMs cells were pre-blocked by each rabbit anti-SRCR domain IgG or RNI and then infected with PRRSV + ICs. The RNA levels (**a**) and the virus titers (**b**) of PRRSV in cell supernatants harvested were evaluated by real-time RT-PCR and TCID_50_ assay. *** *p* < 0.001, ** *p* < 0.01, ns: no significance.

We determined that activating FcγRs were responsible for PRRSV-ADE infection. We next explored if viral receptors, the porcine Sn and CD163 molecules, were required for PRRSV-ADE infection. PAMs cells were pre-blocked with rabbit anti-Sn extracellular N-terminal domain IgG, rabbit anti-CD163 molecule SRCR domain IgG, or RNI for two hours and then infected with PRRSV + ICs. The infected cell supernatants were harvested at different time points for the determination of the RNA and the TCID_50_ of PRRSV. As illustrated in Figure 6, at twelve and twenty-four hours after infection, the RNA of PRRSV and the TCID_50_ of PRRSV in supernatants of PRRSV + ICs-infected PAMs pre-blocked by anti-Sn1, 2, 3, 4, 5, 6, or 1–9 IgG were observably lower than those in supernatants of PRRSV + ICs-infected PAMs pre-blocked by RNI, whereas the RNA of PRRSV and the TCID_50_ of PRRSV in supernatants of PRRSV + ICs-infected PAMs pre-blocked by anti-Sn7, 8, or 9 IgG were no significant differences compared to those in supernatants of PRRSV + ICs-infected PAMs pre-blocked by RNI. As exhibited in Figure 7, at twelve and twenty-four hours after infection, the RNA of PRRSV and the TCID_50_ of PRRSV in supernatants of PRRSV + ICs-infected PAMs pre-blocked by anti-SRCR5 or 1–9 IgG were markedly weaker than those in supernatants of PRRSV + ICs-infected PAMs pre-blocked by RNI, while the RNA of PRRSV and the TCID_50_ of PRRSV in supernatants of PRRSV + ICs-infected PAMs pre-blocked by anti-SRCR1, 2, 3, 4, 6, 7, 8, or 9 IgG were not signally different from those in supernatants of PRRSV + ICs-infected PAMs pre-blocked by RNI. These results showed that specific antibody blocking of Sn1, 2, 3, 4, 5, or 6 domain of porcine Sn protein or selective antibody blocking of SRCR5 domain of porcine CD163 molecule inhibited ADE of PRRSV infection in PAMs cells, which suggested that Sn and CD163 were required for PRRSV-ADE infection.

## 4. Discussion

IgG is a critical part in the humoral immune system. The primary function of IgG is specific recognition and binding of the foreign antigens to generate a complex, then resulting in the losses of the toxicity and the pathogenicity of antigens after the complex is phagocytized, digested, and cleared by immune phagocytes [35,36]. Nevertheless, previous studies have shown that viral propagation may be increased by the formation of virus–antibody ICs, which is called the ADE effect [37]. The phenomenon of ADE of virus infection has been confirmed for several different types of viruses, including PRRSV [6,38]. On the one hand, ADE is an actual cause of the pathogenesis of PRRSV-persistent infection. On the other hand, ADE in PRRSV infection is a significant obstacle to the development of effective PRRS vaccines [11,39]. However, the precise underlying mechanisms connected with ADE of PRRSV infection are still not entirely clear. Fc receptors (FcRs) are key mediators in immune systems linking the innate immune response with adaptive immunity. Unfortunately, FcγRs not only protect the organisms from pathogen infections but also enhance the susceptibility of hosts. The ADE mechanism mediated by FcRs was first demonstrated in the infection of dengue virus [40]. Firstly, the virus and its specific antibodies form infectious virus–antibody ICs. Then, the ICs facilitate the adhesion and endocytosis of the virus to host cells through the FcRs [40,41]. We found that the knockdown of the porcine FcγRI or FcγRIII gene diminished the ADE activity of PRRSV infection in PAMs. Meanwhile, treatment of PAMs cells with FcγRI or FcγRIII-specific IgG to block FcγRI or FcγRIII inhibited enhancement of infection in the presence of PRRSV antibodies (data not shown). These studies suggested that the activating porcine FcγRs were responsible for mediating PRRSV-ADE infection, which was in keeping with the recent reports [16,42]. However, an early study showed that the ADE of HIV-1 infection proceeding via FcγRI required the virus glycoprotein interaction with its cell surface receptor, the CD4 molecule [43]. A recent study demonstrates that the angiotensin-converting enzyme 2 (SARS-CoV-2 receptor) is the secondary receptor required for FcγR-mediated the ADE of SARS-CoV-2 infection [44]. These studies indicate that the natural virus receptors have an important influence on the ADE of virus infection, which provides an intriguing suggestion about the roles of viral receptors in ADE infection by other viruses.

PRRSV infection is a receptor-dependent event. Sn has been widely studied as a crucial protein molecule for PRRSV by binding the virus’s membrane protein/glycoprotein 5 (GP5) complexes [45]. The first N-terminal domain of the porcine Sn extracellular region is a pivotal domain for interaction with the PRRSV [46,47]. Subsequently, another report shows that the first four N-terminal extracellular region domains of porcine Sn are necessary for PRRSV invasion [48]. The CD163 molecule is the other putative cell receptor for PRRSV, and it is indispensable and sufficient to mediate PRRSV infection [49]. The extracellular SRCR5 domain of the CD163 molecule is a vital domain interacting with PRRSV particles [33]. To date, it remains unclear whether the involvement of the Sn and CD163 molecules in ADE of PRRSV infection. A previous study has shown that the viral GP5, a ligand protein for porcine Sn, is closely associated with PRRSV-ADE infection, implying that porcine Sn may have an essential effect on PRRSV-ADE infection [50]. We observed that the treatment of PAMs cells with rabbit anti-Sn1-6 or SRCR5 domain IgG downregulated PRRSV-specific antibody-enhanced PRRSV infection, but the treatment of PAMs cells with rabbit anti-Sn or SRCR other domain-specific IgG had no influence on PRRSV-ADE infection, suggesting that Sn and CD163 molecules were involved in ADE of PRRSV infection. The Sn1-6 extracellular domains of Sn protein and the SRCR5 domain of CD163 molecule were required for PRRSV-ADE infection. The binding of the PRRSV-antibody ICs to FcγRs might accelerate PRRSV particle entry by heightening virus interaction with its receptors (Sn and CD163) expressed on the host cell surface. Nevertheless, the exact mechanism remains to be further elucidated. In future work, we will explore in depth the mechanism of PRRSV-ADE infection mediated by FcγRs and virus receptors. Taken together, FcγR-dependent ADE of PRRSV infection required the participation of the Sn and CD163 molecules. The viral Sn and CD163 receptors played an influential role in PRRSV infection enhancement. The abilities of anti-Sn or CD163 antibodies to block infection enhancement in vitro might have important implications for preventing or decreasing the development of PRRS mediated by antibodies in PRRSV-infected pigs. These studies should allow a more precise understanding of the structural basis for the mechanism of PRRSV-ADE infection, which could be crucial for the development of efficient intervention of PRRSV infection mediated by the antibodies and would provide references for screening targets of novel PRRS vaccines or antiviral drugs against the PRRSV.

## Figures and Tables

**Table 1:** The extracellular domains of porcine Sn protein.

Number	Start Position	End Position
1	26	136
2	145	235
3	253	312
4	337	397
5	418	509
6	522	582
7	609	707
8	714	792
9	807	882
10	906	966
11	995	1073
12	1091	1169
13	1184	1248
14	1272	1331
15	1358	1433
16	1457	1519
17	1562	1637

**Table 2:** The SRCR domains of porcine CD163 molecule.

Name	Start Position	End Position
SRCR1	51	151
SRCR2	158	258
SRCR3	265	365
SRCR4	372	472
SRCR5	477	577
SRCR6	582	682
SRCR7	718	818
SRCR8	823	925
SRCR9	928	1028

**Table 3:** The sequences of siRNAs.

Gene Name	Sequence (5′-3′)
Porcine FcγRI	Forward: GCCUUGAGGUGUCAUGGAUTT Reverse: AUCCAUGACACCUCAAGGCTT
Porcine FcγRIII	Forward: GUGGAGAAUACACGUGUAATT Reverse: UUACACGUGUAUUCUCCACTT
Negative control	Forward: UUCUCCGAACGUGUCACGUTT Reverse: ACGUGACACGUUCGGAGAATT

**Table 4:** The sequences of relative quantitative RT-PCR primers.

Name	Sequence (5′-3′)
Porcine FcγRI forward Porcine FcγRI reverse	TGAAACAAAGTTGCTCCCA GCTGCGCTTGATGACCT
Porcine FcγRIII forward Porcine FcγRIII reverse	CTGCTGCTTCTGGTTTCA CCATTCCACCTCCACTC
β-actin forward β-actin reverse	CGGGACATCAAGGAGAAGC CTCGTTGCCGATGGTGATG

## Data Availability

The available data are found in the paper.

## Supplementary Materials

The following supporting information can be downloaded at: https://www.mdpi.com/article/10.3390/vetsci9090470/s1, Figure S1 FcR1-48h 2019.04.02_08.04.47_Ch+Marker; Figure S2 FCR3-48H-2018.08.20_08.03.12_Ch+Marker; Figure S3 gapdh-48h 2019.04.02_08.01.16_Ch+Marker; Figure S4 gapdh-fc3-48h-3 2019.05.10_06.45.27_Ch+Marker.
