# Supplementary figures and images for: Activating Fc Gamma Receptors and Viral Receptors Are Required for Antibody-Dependent Enhancement of Porcine Reproductive and Respiratory Syndrome Virus Infection
Source: Vet Sci. 2022 Aug 31;9(9):470. doi: 10.3390/vetsci9090470 (PMC9504219; doi:10.3390/vetsci9090470)

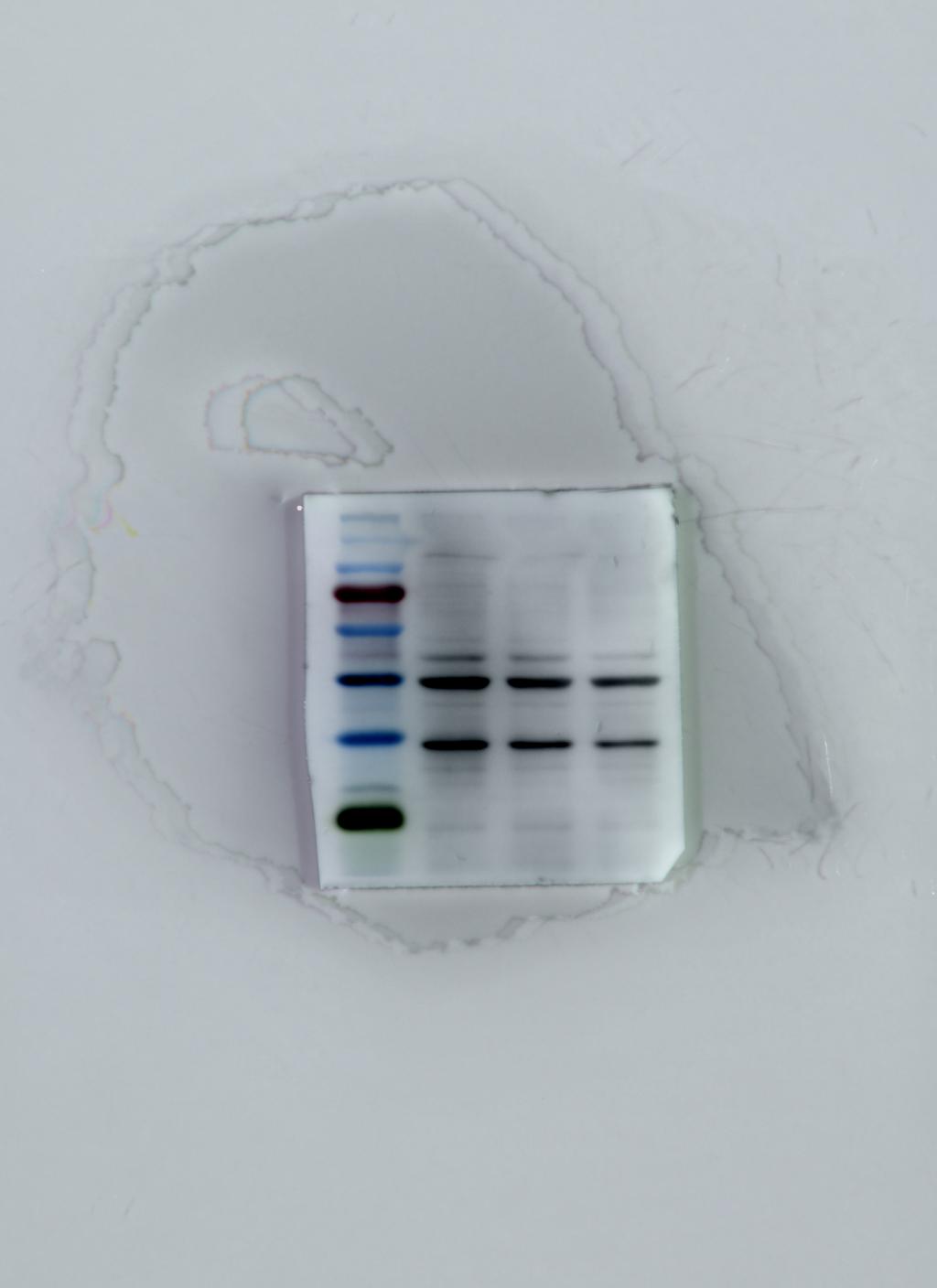

Supplement: Supplementary file 1 [file vetsci-09-00470-s001.zip › Figure S1 FcR1-48h 2019.04.02_08.04.47_Ch+Marker.jpg]

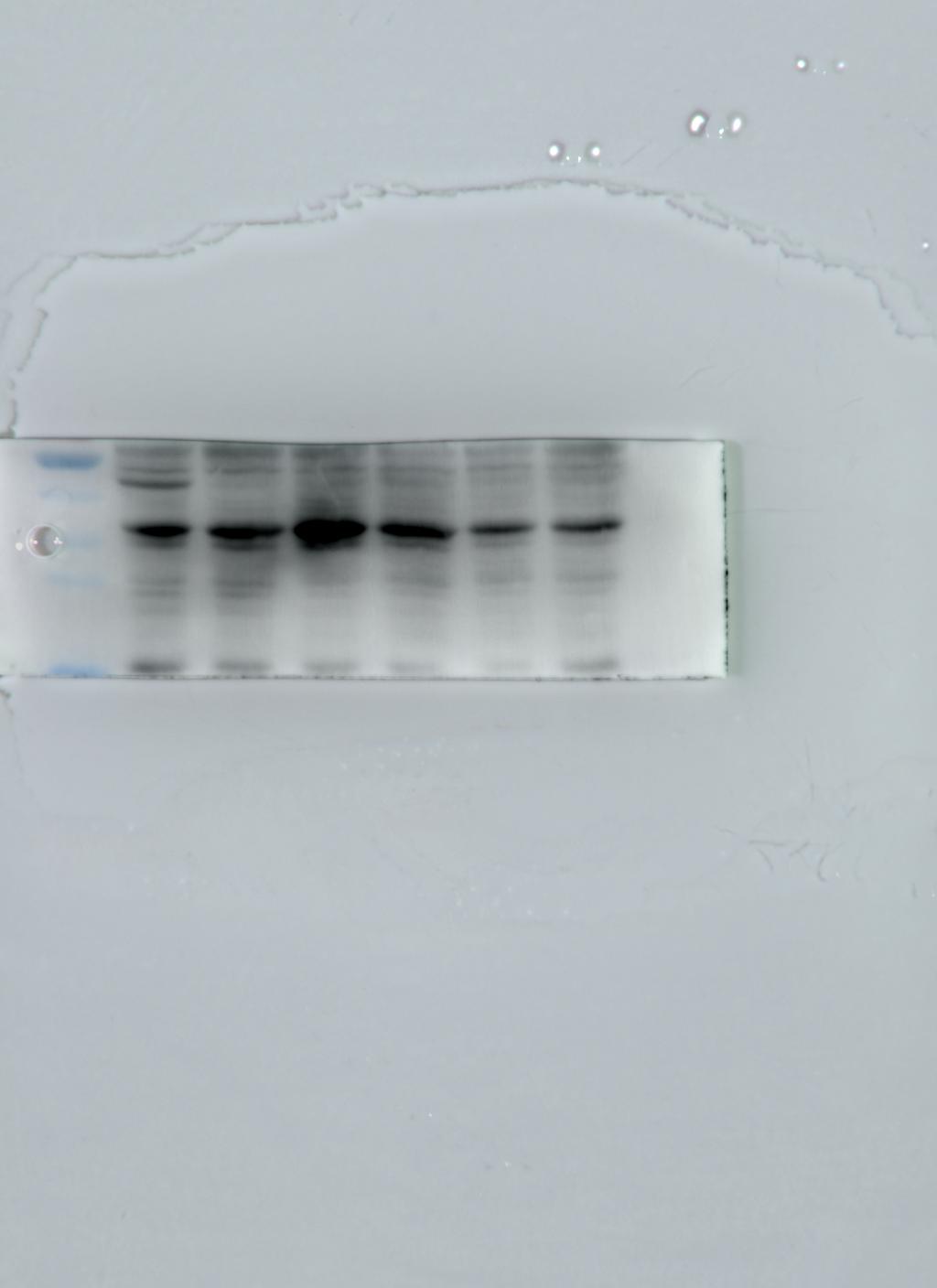

Supplement: Supplementary file 1 [file vetsci-09-00470-s001.zip › Figure S2 FCR3-48H-2018.08.20_08.03.12_Ch+Marker.jpg]

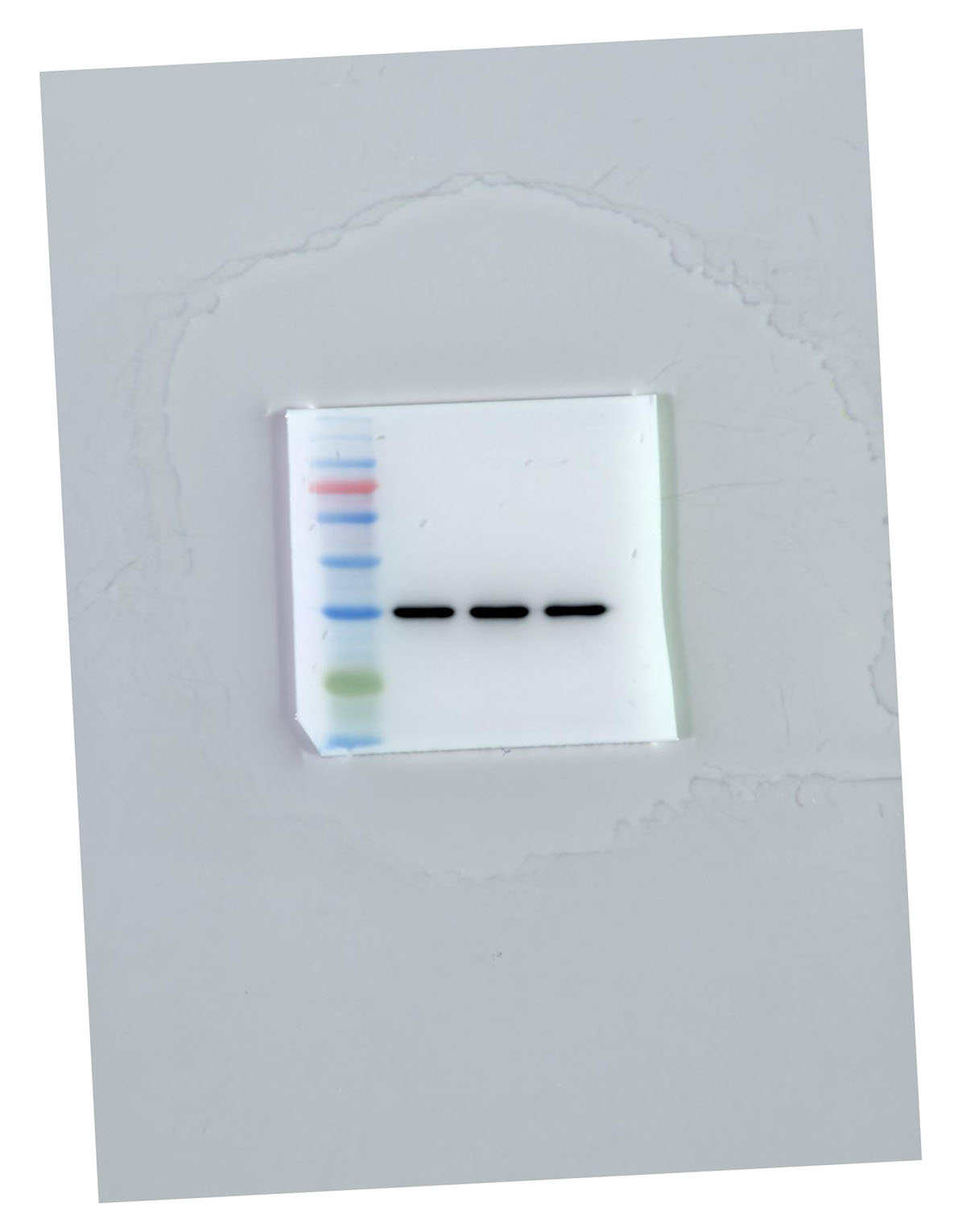

Supplement: Supplementary file 1 [file vetsci-09-00470-s001.zip › Figure S3 gapdh-48h 2019.04.02_08.01.16_Ch+Marker.jpg]

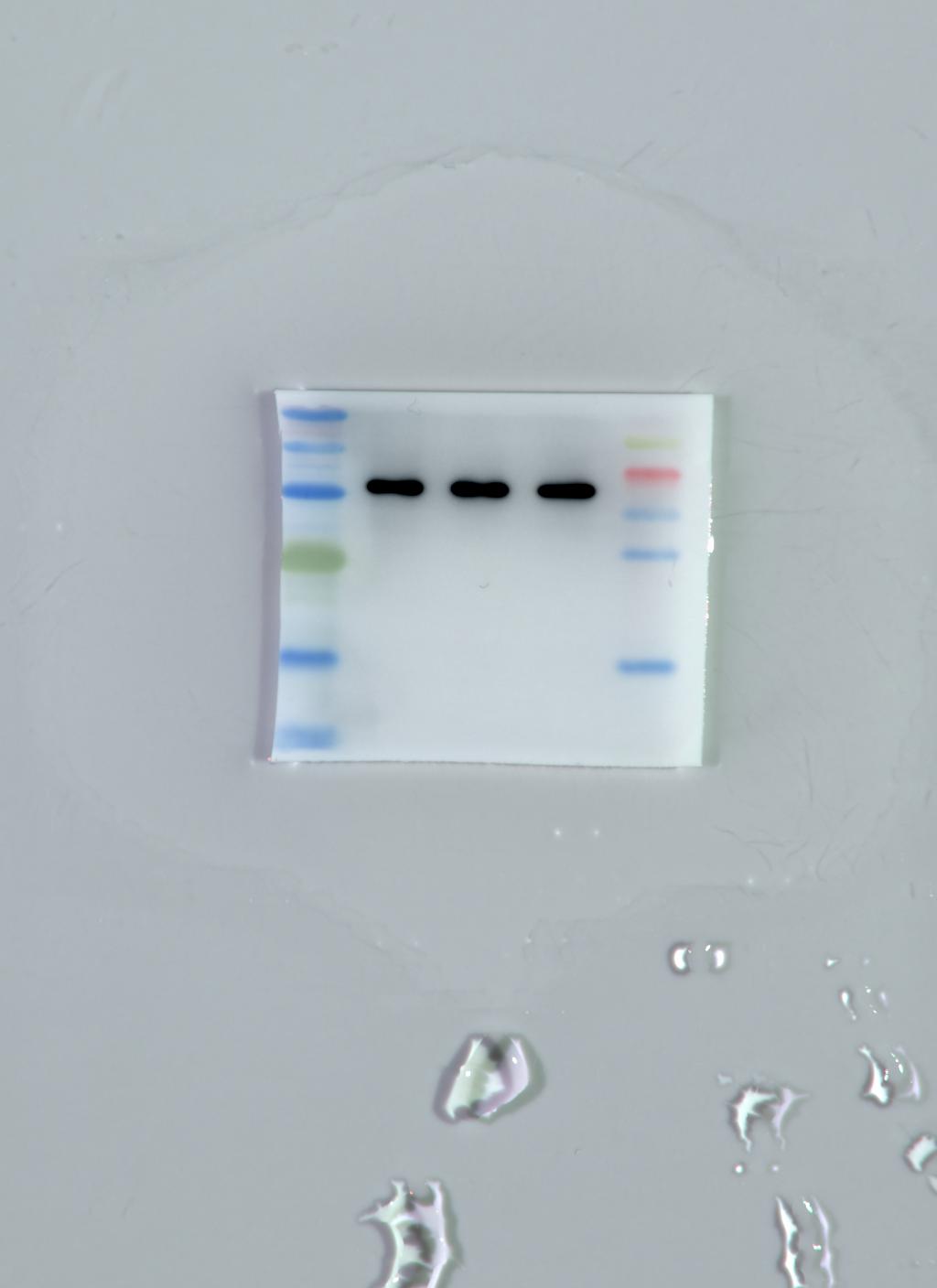

Supplement: Supplementary file 1 [file vetsci-09-00470-s001.zip › Figure S4 gapdh-fc3-48h-3 2019.05.10_06.45.27_Ch+Marker.jpg]
